# The Importance of a Definite Diagnosis for Rational Treatment and Prognosis of Head and Neck Tumors†

**DOI:** 10.3390/cancers17142311

**Published:** 2025-07-11

**Authors:** Alfio Ferlito, Alfons Nadal, Göran Stenman, Nina Zidar, Henrik Hellquist, Piet J. Slootweg, Roderick H. W. Simpson, Antonio Cardesa

**Affiliations:** 1 International Head and Neck Scientific Group, 35100 Padua, Italy; profalfioferlito@gmail.com; 2 Department of Pathology, Hospital Clinic, 08036 Barcelona, Spain; anadal@clinic.cat; 3 Department of Basic Clinical Practice, School of Medicine, Universitat de Barcelona, 08036 Barcelona, Spain; 4 Institut d’Investigacions Biomèdiques August Pi i Sunyer, 08036 Barcelona, Spain; 5 Department of Laboratory Medicine/Pathology, Sahlgrenska Center for Cancer Research, University of Gothenburg, Sahlgrenska University Hospital, SE-405 30 Gothenburg, Sweden; goran.stenman@gu.ser; 6 Faculty of Medicine, Institute of Pathology, University of Ljubljana, Korytkova 2, 1000 Ljubljana, Slovenia; Nina.ZIDAR@mf.uni-lj.si; 7 Department of Biomedical Sciences and Medicine, ABC-RI, University of Algarve, 8005-139 Faro, Portugal; henrikhellquist@pm.me; 8 Department of Cellular Pathology, Northern Lincolnshire and Goole NHS Foundation Trust, Lincoln LN2 5QY, UK; 9 Radboud University Medical Center, 6511HB Nijmegen, The Netherlands; piet.slootweg@radboudumc.nl; 10 Kilimanjaro Christian Medical University College, Moshi 2240, Tanzania; 11 Department of Anatomical Pathology, University of Calgary, Calgary, AB T2N 2T9, Canada; roderick.simpson@doctors.org.uk; 12 Universitat de Barcelona, 08036 Barcelona, Spain

Benign and malignant head and neck tumors are common worldwide and cause mortality and morbidity with variations in population prevalence. Obviously, a correct diagnosis is of paramount importance for choosing the appropriate treatment and offering a correct prognosis.

There are many types of diagnoses, such as provisional or working diagnosis, diagnosis by exclusion, diagnosis ex juvantibus, provocative diagnosis, direct diagnosis, deductive diagnosis, physical diagnosis, laboratory diagnosis, differential diagnosis, clinical diagnosis, cytologic diagnosis, frozen-section diagnosis, molecular diagnosis, pathologic diagnosis, and final or permanent section diagnosis [1]. In particular, a qualitative diagnosis is made by a pathologist, whereas a quantitative diagnosis, such as the identification of a mass lesion and the estimation of tumor volume using 3D imaging, is provided by a radiologist using modern imaging techniques, including CT or MRI.

To avoid mismanagement, recurrences, and complications for the patient, it is essential that the correct diagnosis is established prior to commencement of the definitive treatment. Therefore, a histopathologic diagnosis, supported by immunohistochemistry and molecular analyses when required, is indicated, although it should be noted that these additional techniques are not always available everywhere due to the costs involved and the necessary facilities. Moreover, histopathologic examination enables the detection of, for example, HPV- and EBV-associated tumors, allowing for optimal treatment, which may be different from their virus-negative counterparts.

Some examples could be useful to further clarify this issue. Radiological identification of a mass in the larynx as a possible chondrosarcoma is not sufficient to indicate the correct treatment. For conventional chondrosarcomas, surgical treatment is confined to the larynx. However, if the tumor is a dedifferentiated or myxoid chondrosarcoma, surgery is the mainstay of therapy, and total laryngectomy with radical intent is the most common choice. Radiotherapy may be used as an adjuvant in this setting, even though chondrosarcomas are classically considered radioresistant tumors. Similarly, dedifferentiated chondrosarcomas seem to respond better to chemotherapy compared to their less aggressive counterparts. When comparing the prognosis and survival rates of G3 dedifferentiated and myxoid chondrosarcomas to those of G1 and G2 lesions, they have a poorer prognosis, with higher rates of recurrences and locoregional and distant metastases even after radical surgery [2]. Obviously, the diagnosis of these aggressive subtypes of chondrosarcomas must be pathological and not only radiological.

A radiological diagnosis of lipoma could prove inaccurate after histopathologic examination and turn into a well-differentiated liposarcoma. Similarly, cytological examination of a salivary gland mass may suggest a pleomorphic adenoma, whereas the final histopathological diagnosis is a malignant tumor (e.g., adenoid cystic carcinoma, myoepithelial carcinoma, polymorphous adenocarcinoma, or mucoepidermoid carcinoma) [3]. A clinical diagnosis of an aural polyp of the middle ear mucosa could be hiding a rhabdomyosarcoma disclosed in the final histopathological diagnosis [4]. Exceptionally, a clinical diagnosis of adenoids may lead to a histopathological diagnosis of rhabdomyosarcoma (AF, personal observation). Clinically and radiologically benign mucosal cysts can, on very rare occasions, receive a diagnosis of low-grade mucoepidermoid carcinoma by histopathology (HH, personal observation). Treatment decisions in patients with neoplasms should always be based on a histopathological diagnosis. Radiology can provide useful information regarding the cystic nature, infiltrative pattern, or vascular component of the lesions and may also identify cartilaginous neoplastic lesions. However, it has limitations in distinguishing benign from low-grade malignant chondroid lesions in the absence of aggressive features. There are no consistent, reproducible radiographic criteria to permit differentiation between chondrosarcoma and chondroma [5]. However, it may be demanding or even impossible to differentiate between a chondroma and a low-grade chondrosarcoma even by histopathology. It should also be noted that chondromas may contain cartilaginous areas, which can lead to them being mistaken for chondrosarcoma [6], particularly on small biopsies. Moreover, rapidly growing fibro-osseous lesions of the jaws may be mistaken for osteosarcoma [6].

In terms of working practice, the need for knowledge, communication, transparency, and competency is of paramount importance in making an accurate diagnosis, but as humans, we remain fallible [1].
